# Supplementary figures and images for: Adeno-associated virus type 2 (AAV2) uncoating is a stepwise process and is linked to structural reorganization of the nucleolus
Source: PLoS Pathog. 2022 Jul 11;18(7):e1010187. doi: 10.1371/journal.ppat.1010187 (PMC9302821; doi:10.1371/journal.ppat.1010187)

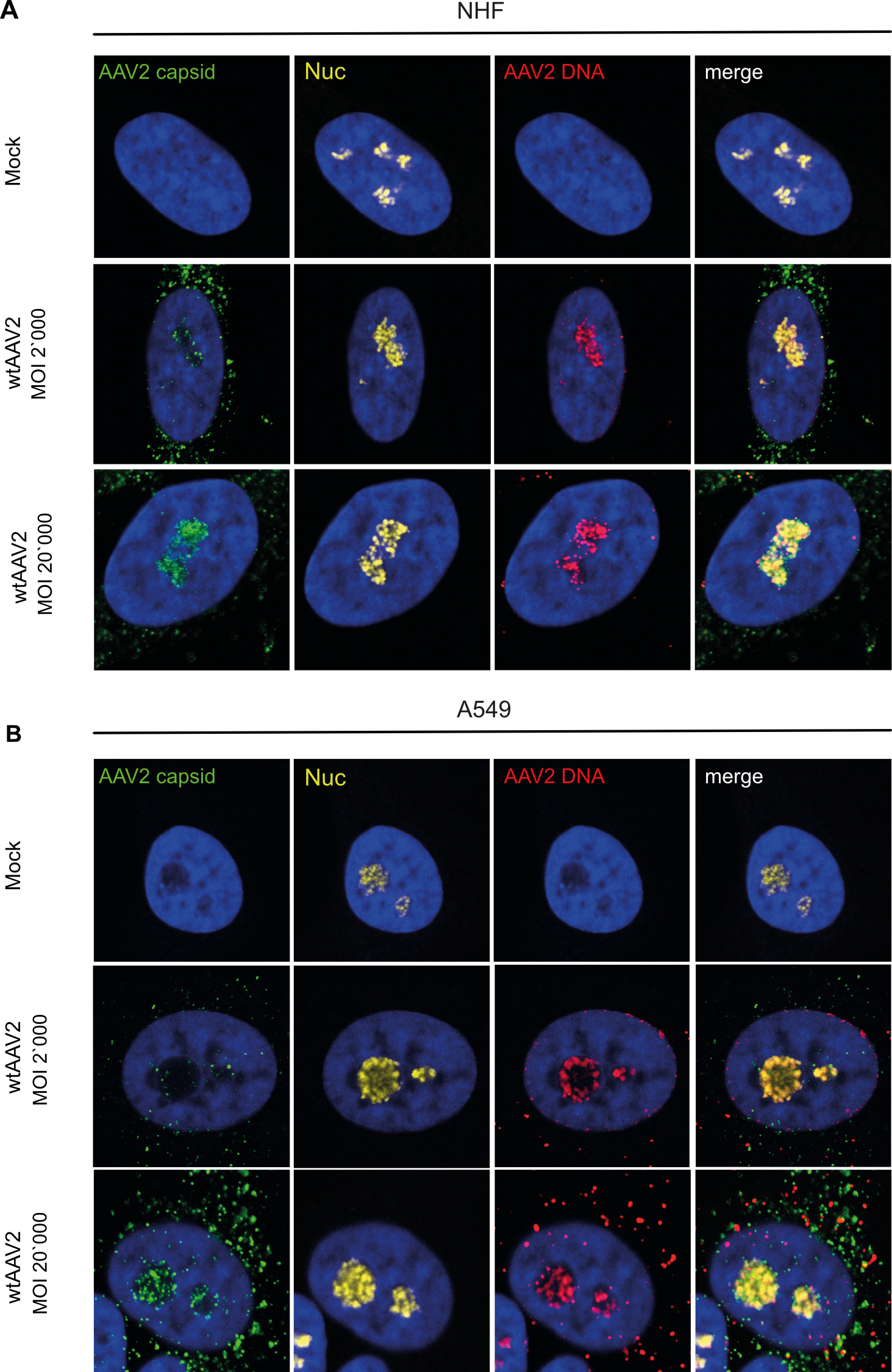

Supplement: S1 Fig — (A) NHF or (B) A549 cells were either mock-infected of infected with wtAAV2 at a MOI of 20`000 or 2`000. At 24 hpi, the samples were processed for IF-FISH and CLSM. Intact capsids were stained using an antibody that detects a conformational capsid epitope (green). Nucleoli (Nuc) were visualized using an antibody against fibrillarin (yellow). AAV2 DNA (red) was detected with an Alexa Fluor (AF) 647 labeled, amine-modified DNA probe that binds to the AAV2 genome. Nuclei were counterstained with DAPI (blue). (TIF) [file ppat.1010187.s001.tif]

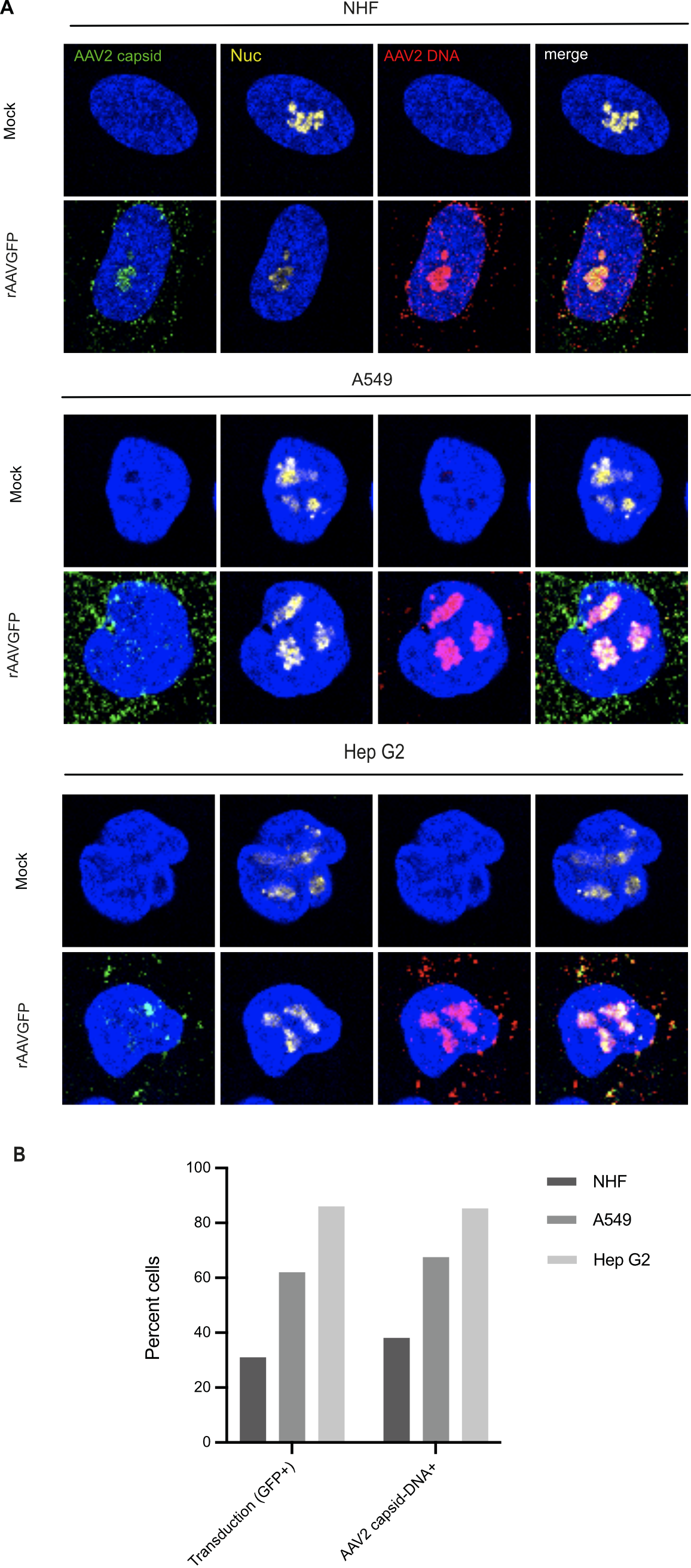

Supplement: S2 Fig — NHF, A549 or Hep G2 cells were either mock-infected or infected with a recombinant AAV2 vector (rAAVGFP) at a MOI of 20`000. At 24 hpi, the samples were processed for IF-FISH and CLSM. (A) Intact capsids were stained using an antibody that detects a conformational capsid epitope (green). Nucleoli (Nuc) were visualized using an antibody against fibrillarin (yellow). AAV2 DNA (red) was detected with an Alexa Fluor (AF) 647 labeled, amine-modified DNA probe that binds to the AAV2 genome. Nuclei were counterstained with DAPI (blue). (B) Comparison of transduction efficiency (% GFP positive cells) and uncoating efficiency (% rAAV capsid-negative DNA-positive nucleoli) of at least 50 cells. (TIFF) [file ppat.1010187.s002.tiff]

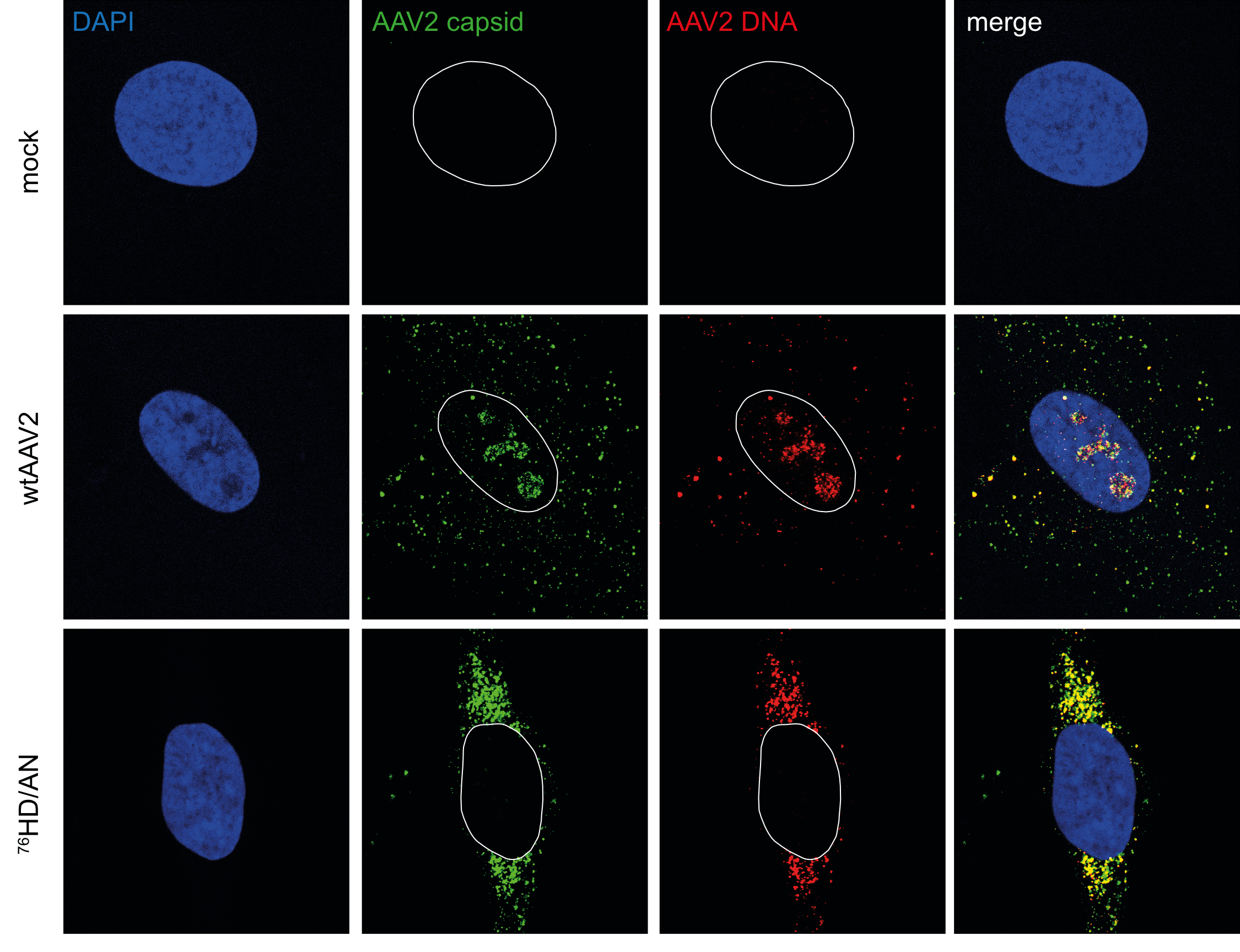

Supplement: S3 Fig — NHF cells were either mock-infected or infected with wtAAV2 or a VP1 AAV2 mutant (76HD/AN) at a MOI of 20`000. At 5 hpi, the samples were processed for IF-FISH and CLSM. Intact capsids were stained using an antibody that detects a conformational capsid epitope (green). AAV2 DNA (red) was detected with an Alexa Fluor (AF) 647 labeled, amine-modified DNA probe that binds to the AAV2 genome. Nuclei were counterstained with DAPI (blue) or illustrated as white lines. (TIF) [file ppat.1010187.s003.tif]

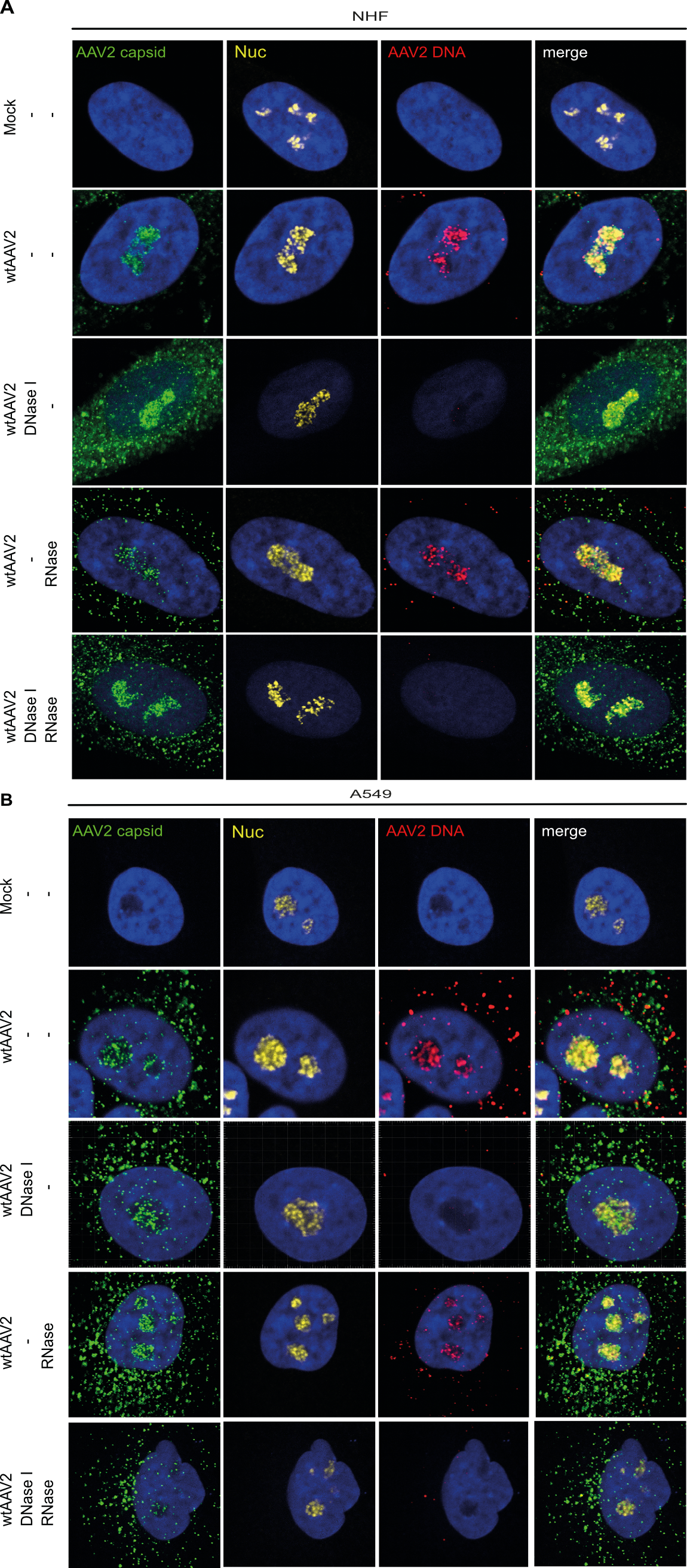

Supplement: S4 Fig — (A) NHF or (B) A549 cells were infected with wtAAV2 (MOI 20`000). At 24 hpi, the cells were either treated with DNase I (1 U/μl) for 1 h at 37°C, RNase A (0.5 mg/ml) for 1 h at 37°C, or a combination of both. DNase or RNase inactivation was achieved by washing the cells twice for at least 10 min with either DNase inactivation buffer (30% Formamide, 0.1% Triton X-100, 2X SSC) or RNase inactivation buffer (0.5X SSC, 0.1 SDS), respectively. Afterwards the samples were fixed and processed for multicolor IF analysis combined with FISH and CLSM. Intact capsids were stained using an antibody that detects a conformational capsid epitope (green). Nucleoli (Nuc) were visualized using an antibody against fibrillarin (yellow). AAV2 DNA (red) was detected with an Alexa Fluor (AF) 647 labeled, amine-modified DNA probe that binds to the AAV2 genome. Nuclei were counterstained with DAPI. (TIF) [file ppat.1010187.s004.tif]

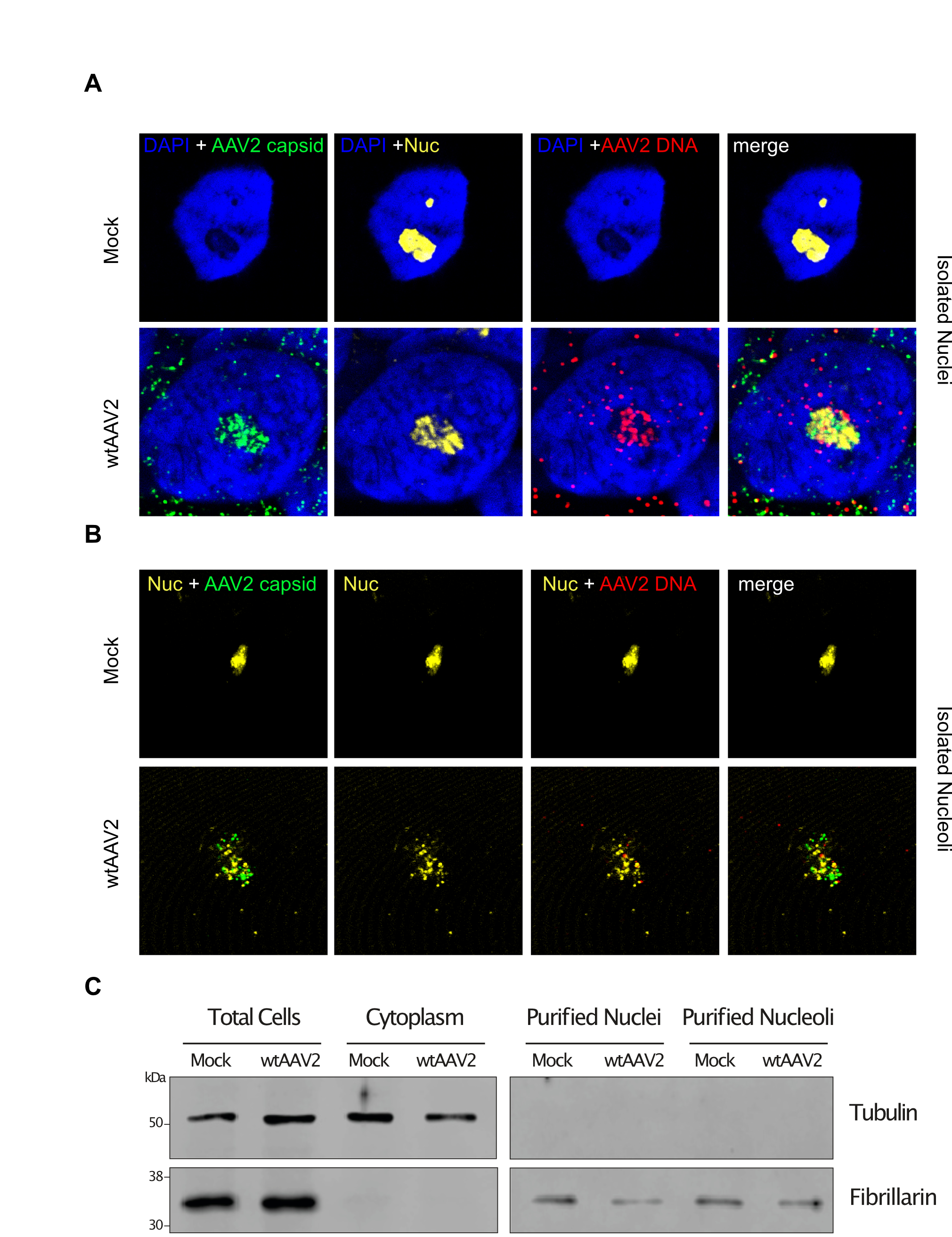

Supplement: S5 Fig — NHF cells were either mock-infected or infected with wtAAV2 at an MOI of 20`000. At 24 hpi, the cells were fractionated and isolated nuclei (A) or nucleoli (B) were applied to fibronectin coated coverslips and processed for IF-FISH and CLSM for morphological assessment of the two fractions. Intact capsids were stained using an antibody that detects a conformational capsid epitope (green). Nucleoli (Nuc) were visualized using an antibody against fibrillarin (yellow). AAV2 DNA (red) was detected with an Alexa Fluor (AF) 647 labeled, amine-modified DNA probe that binds to the AAV2 genome. Nuclei were counterstained with DAPI (blue). (C) Western analysis revealed the absence of α-tubulin (cytoplasmic marker) and presence of fibrillarin (nuclear and nucleolar marker) in the nuclear and nucleolar fractions (105 cell equivalents per lane were loaded). (TIF) [file ppat.1010187.s005.tif]

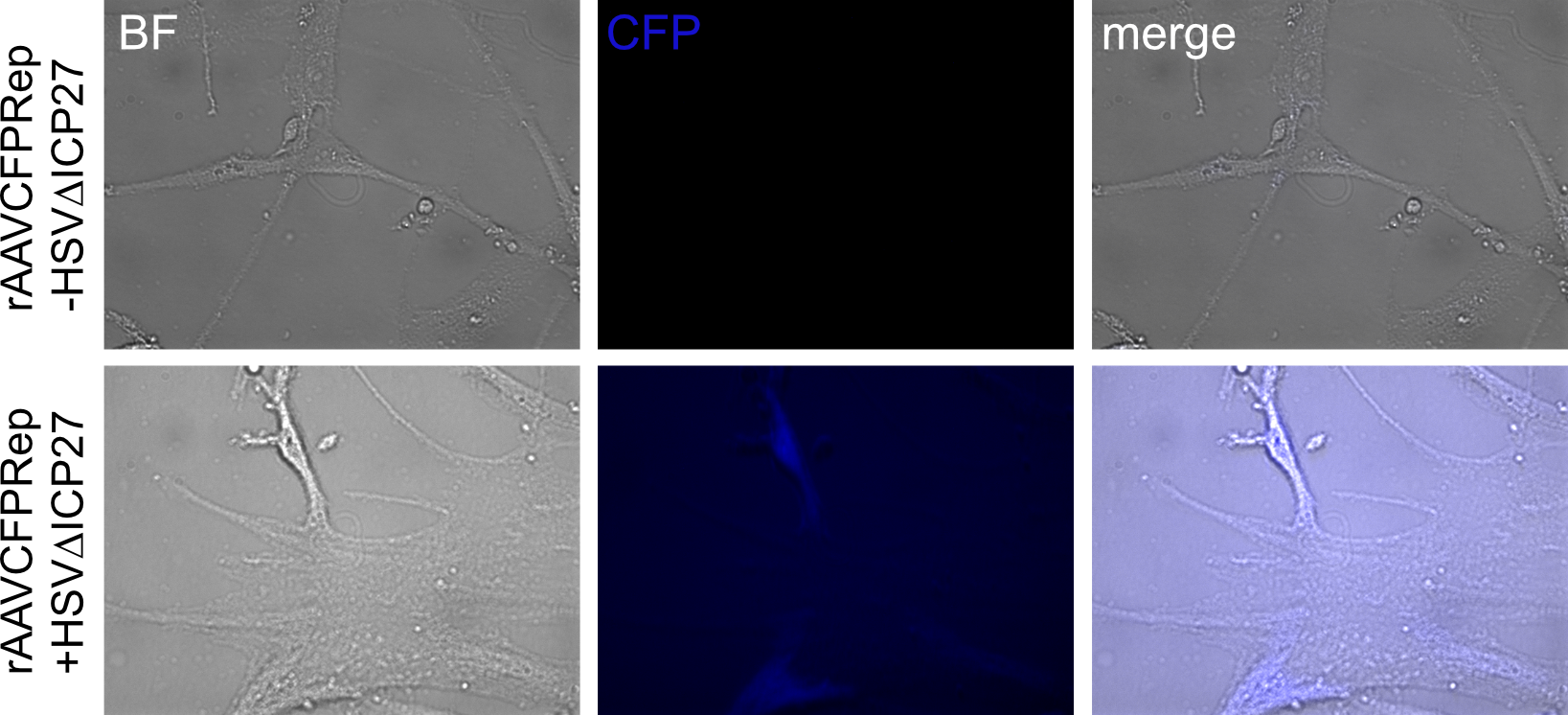

Supplement: S6 Fig — NHF cells were infected with rAAVCFPRep (MOI 20’000), and nucleolar fractions were prepared 24 h later. Bead-purified (uncoated) nucleolar DNA was transfected into NHF cells and, after 24 h, the cells were mock-infected or infected with HSV-1ΔICP27. At 48 hpi, the cultures were monitored for CFP-fluorescence using an epifluorescence microscope. (TIFF) [file ppat.1010187.s006.tiff]

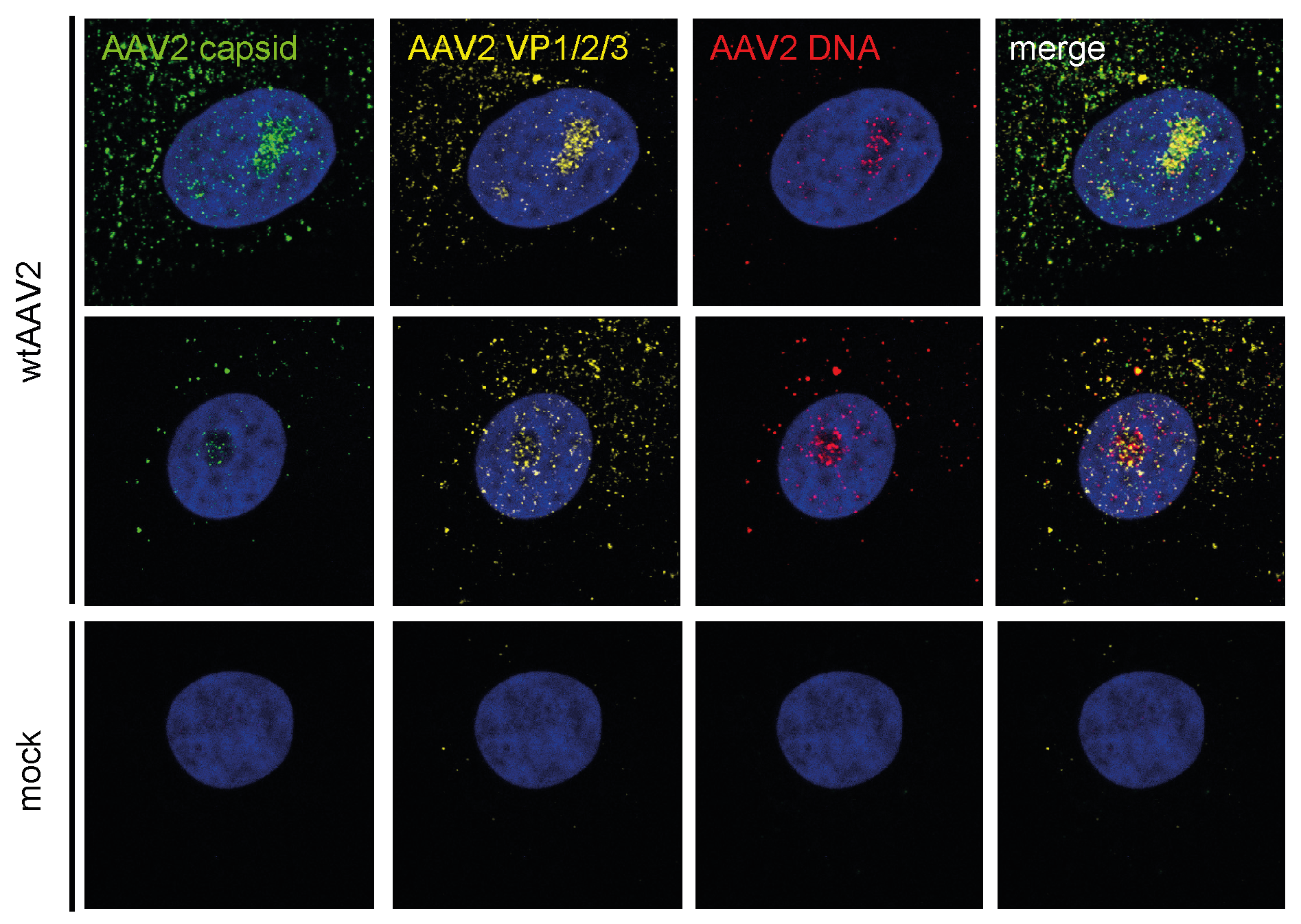

Supplement: S7 Fig — Vero cells were mock-infected or infected with wtAAV2 (MOI 20`000; two individual cells are shown). At 24 hpi, the cells were fixed and processed for multicolor IF analysis combined with FISH and CLSM. Intact capsids (green) or capsid proteins (yellow) were detected using either an antibody against intact AAV2 capsids (conformational capsid epitope) or an antibody (linear epitope) against VP1, VP2 and VP3. AAV2 DNA (red) was detected with an Alexa Fluor (AF) 647 labeled, amine-modified DNA probe that binds to the AAV2 genome. Nuclei were counterstained with DAPI (blue). (TIF) [file ppat.1010187.s007.tif]

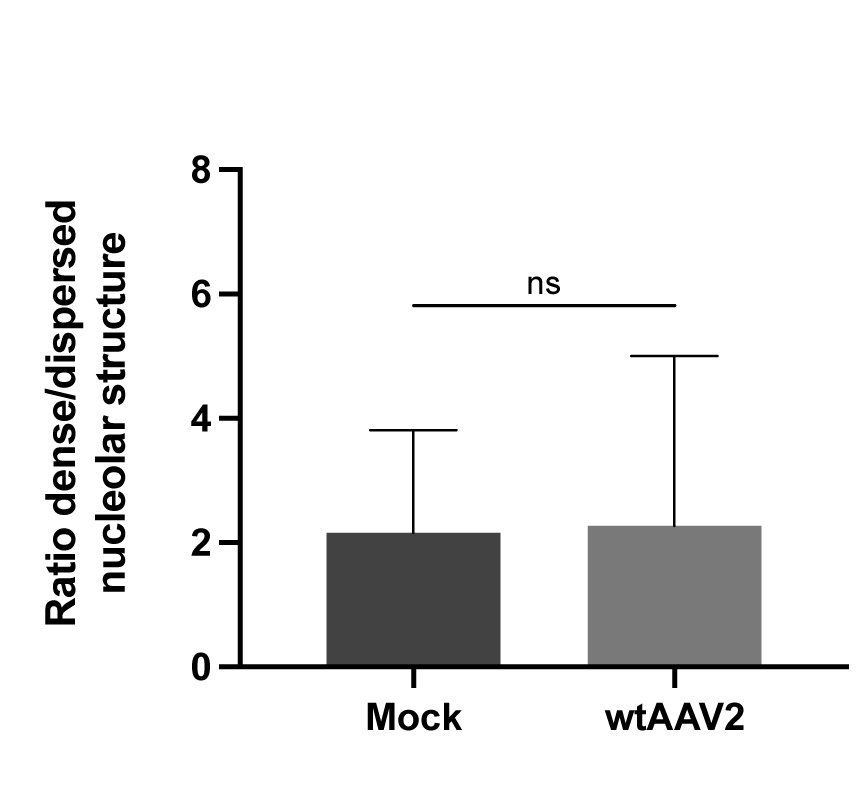

Supplement: S8 Fig — NHF cells were mock-infected or infected with wtAAV2 (MOI 20`000) and 24 h later processed for combined IF-FISH, CLSM and quantification of the nucleolar structure of 100 individual nuclei in mock- or AAV2 infected cells. p-values were calculated using an unpaired Student`s t-test (*—p ≤ 0.05, **—p ≤ 0.01, ***—p ≤ 0.001, ****—p ≤ 0.0001). (TIF) [file ppat.1010187.s008.tif]

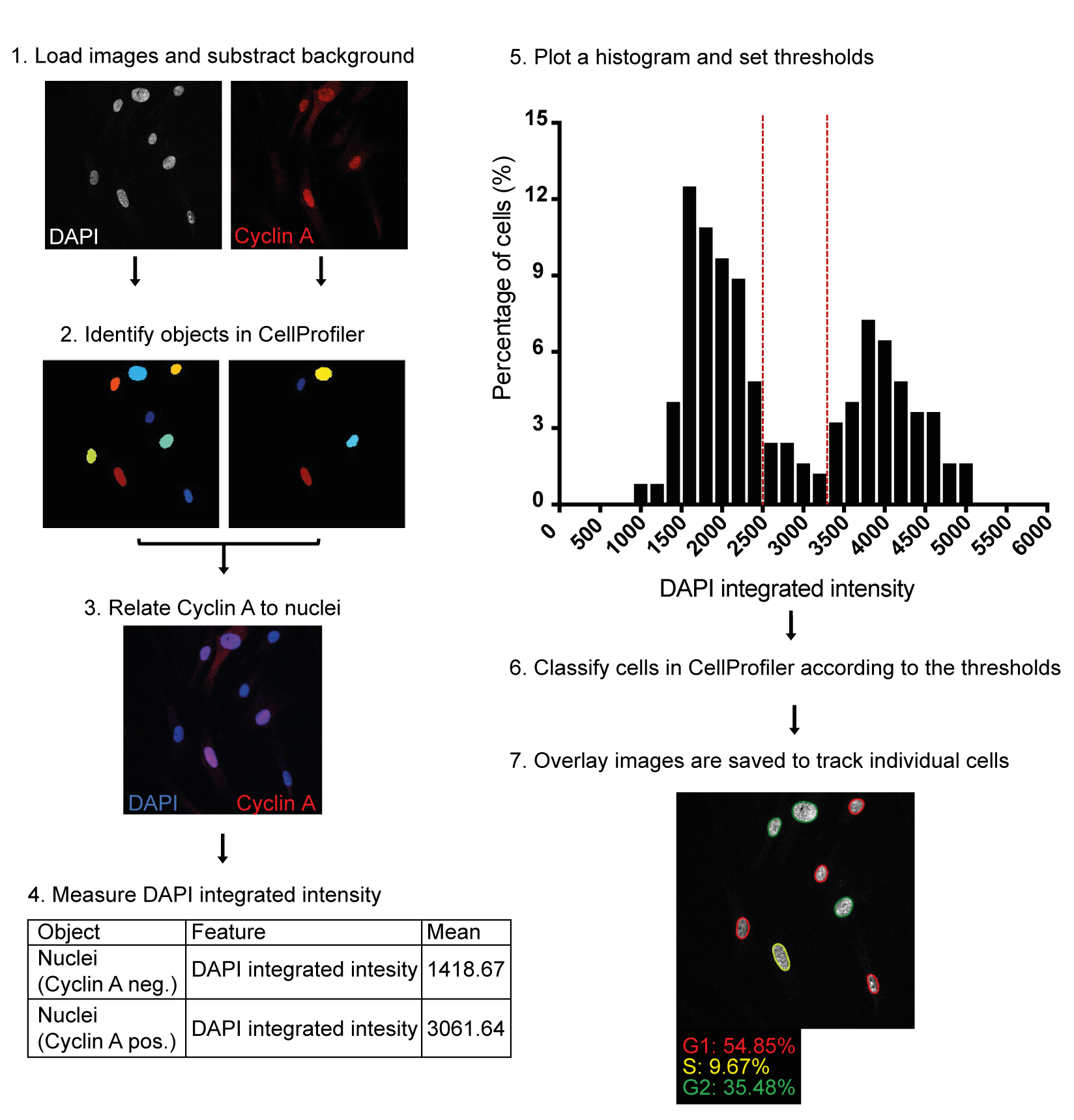

Supplement: S9 Fig — (1) The background of each image was subtracted. (2) Nuclei and cyclin A stainings were identified as primary objects in CellProfiler. (3) The stainings were related to each other and (4) the DAPI integrated intensity of each nucleus was measured. (5) Histograms of the DAPI integrated intensities were plotted using an automated script in Matlab and visual thresholds were set. (6) Cells were classified in CellProfiler using the visual thresholds obtained in step 5. (7) The classification of each nucleus into G1, S or G2 was overlayed on the original DAPI image to track individual cells. (TIF) [file ppat.1010187.s009.tif]

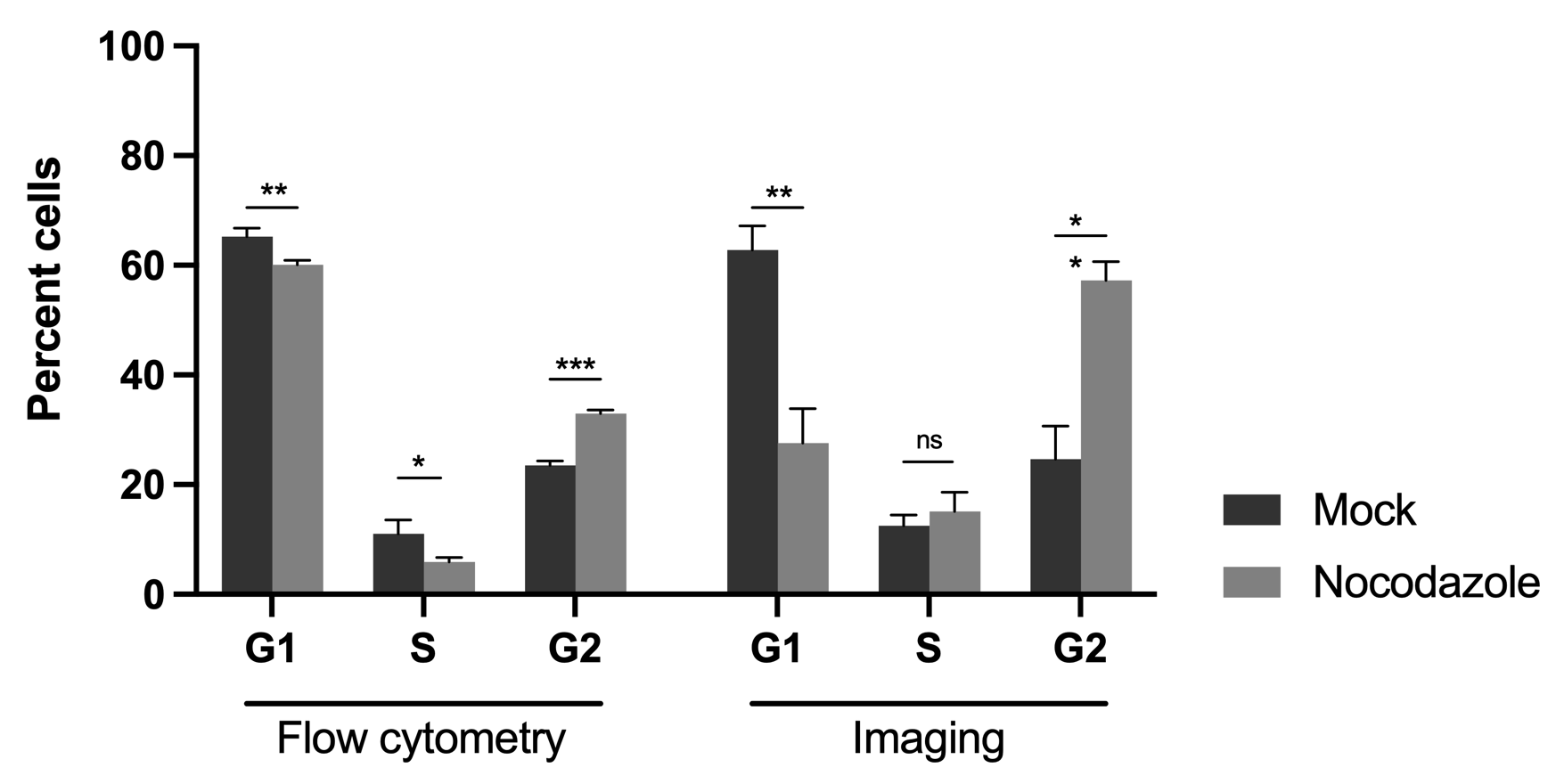

Supplement: S10 Fig — NHF cells were synchronized using a double thymidine (3 mM) block. After the release, the cells were either mock-treated or treated with nocodazole (200 nM) for 24 hours to induce a G2 cell cycle phase arrest. Flow cytometry shows the mean value of three experiments, each replicate contains at least 5`000 scored events. CLSM analysis shows the mean value of three experiments, each replicate contains at least 100 individual analyzed cells. p-values were calculated using an unpaired Student`s t-test (*—p ≤ 0.05, **—p ≤ 0.01, ***—p ≤ 0.001, ****—p ≤ 0.0001). (TIFF) [file ppat.1010187.s010.tiff]

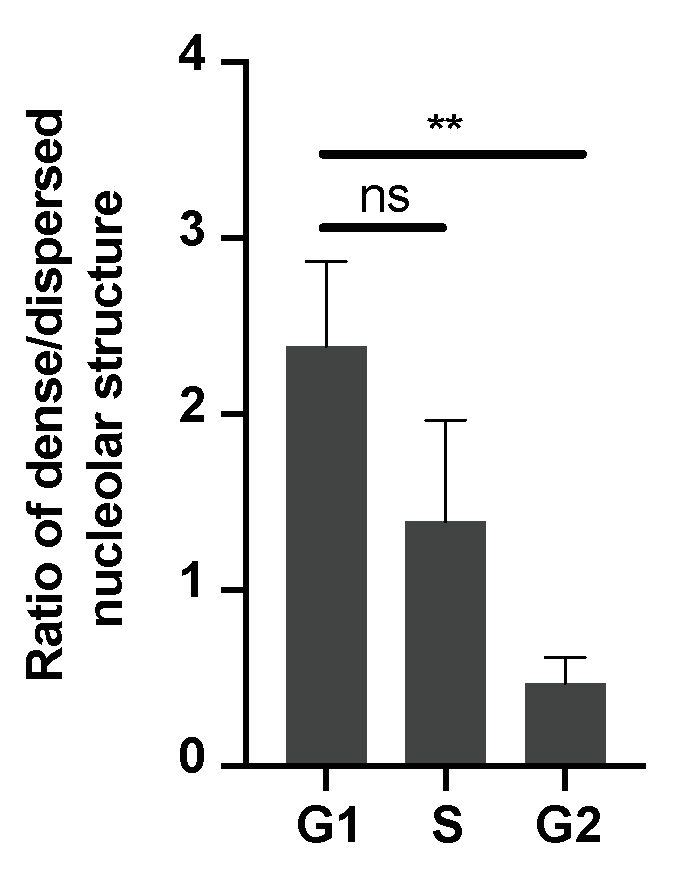

Supplement: S11 Fig — Image-based analysis of the ratios of dense to dispersed nucleoli of 150 individual cells in different cell cycle phases (G1, S and G2). Statistical analysis was performed on three independent experiments and p-values were calculated using an unpaired Student`s t-test (*—p ≤ 0.05, **—p ≤ 0.01, ***—p ≤ 0.001, ****—p ≤ 0.0001). (TIF) [file ppat.1010187.s011.tif]

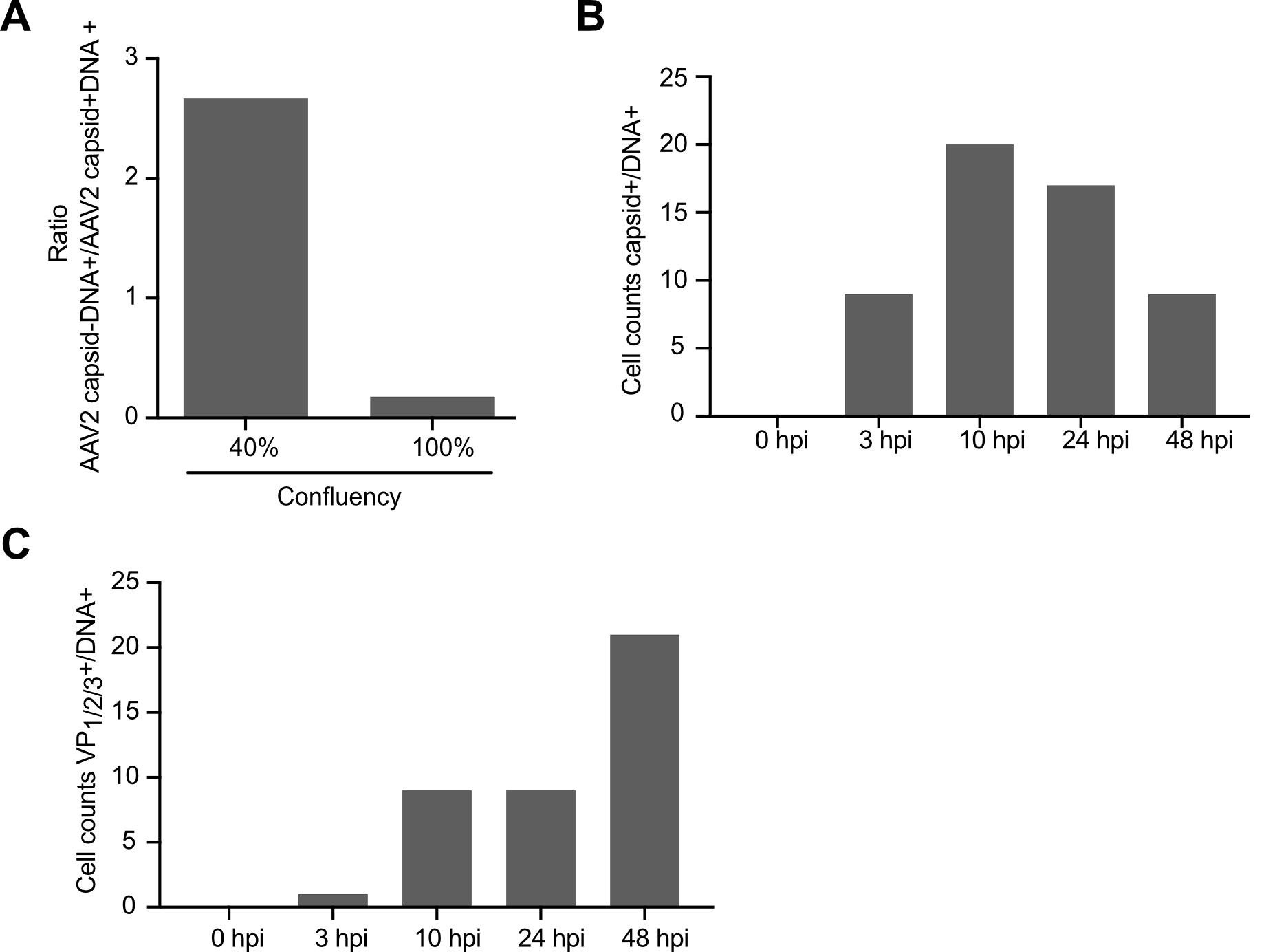

Supplement: S12 Fig — NHF cells were seeded at 40% or 100% confluency, respectively. The cells were either mock-infected or infected with wtAAV2 (MOI 20’000) and, at various time points post infection, processed for combined IF-FISH and CLSM. (A) Image-based analysis of the total uncoating rate in nucleoli of 30 cells at 48 hpi at 40% and 100% confluency. Quantification of cells (30 cells per time point) with (B) AAV2 capsid-positive and DNA-positive (AAV2 capsid+DNA+) signal or (C) AAV2 capsid protein VP1, VP2 and VP3-positive and DNA-positive (AAV2 VP1/2/3+DNA+) signal in nucleoli. (TIFF) [file ppat.1010187.s012.tiff]

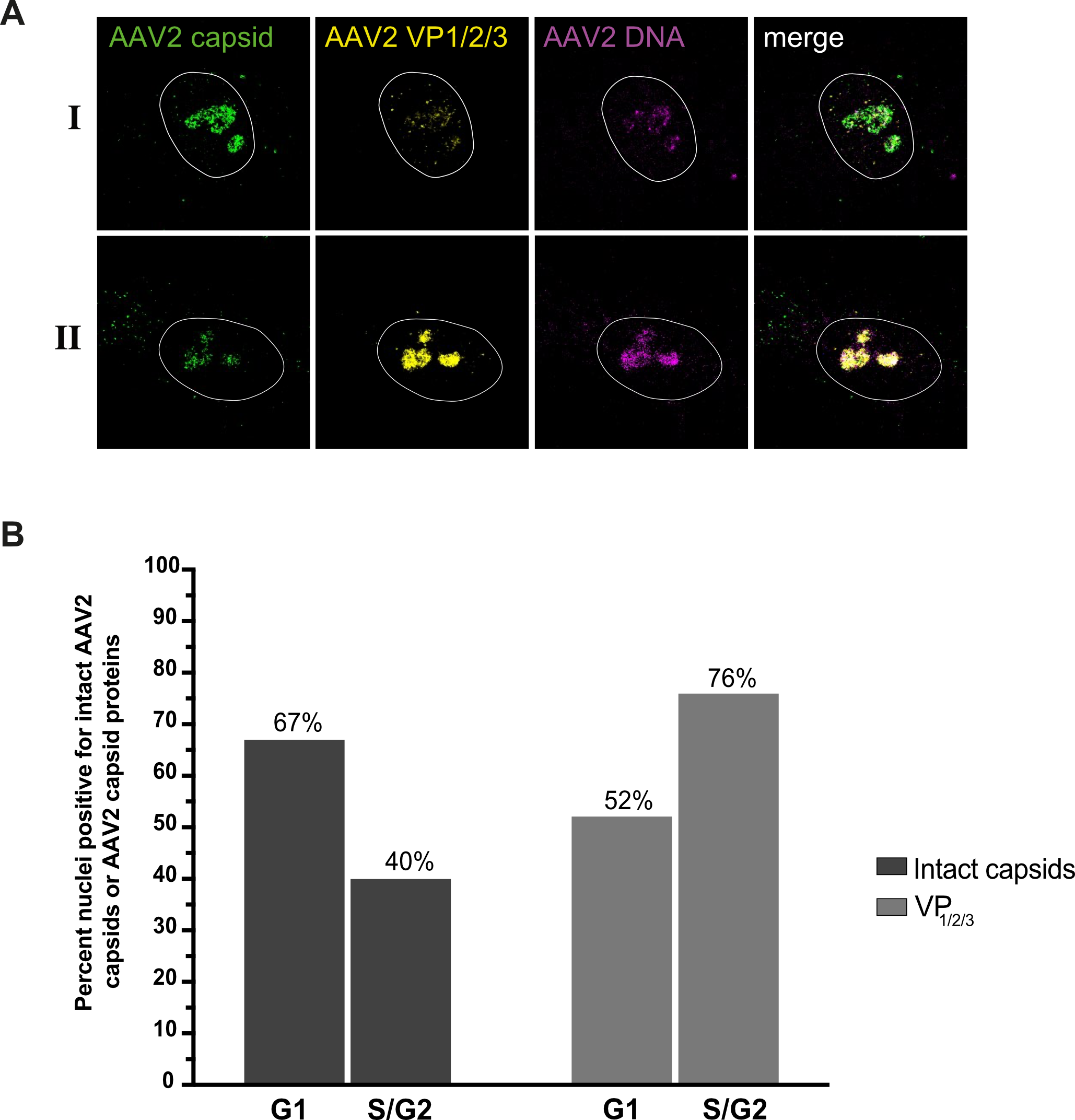

Supplement: S13 Fig — HDFn cells were infected with wtAAV2 (MOI 20`000). At 24 hpi, the cells were fixed and processed for multicolor IF analysis combined with FISH, CLSM. (A) Intact capsids (green) or capsid proteins (yellow) were detected using either an antibody against intact AAV2 capsids (conformational capsid epitope) or an antibody (linear epitope) against VP1, VP2 and VP3. AAV2 DNA (magenta) was detected with an Alexa Fluor (AF) 647 labeled, amine-modified DNA probe that binds to the AAV2 genome. Nuclei were counterstained with DAPI and illustrated as white lines. The wtAAV2 infected cells shown were assigned as pattern I and II according to the intensity of the capsid staining and following a similar classification as defined in Fig 4. (B) Quantification of at least 50 nuclei positive for intact AAV2 capsids or capsid proteins during cell cycle progression. (TIFF) [file ppat.1010187.s013.tiff]

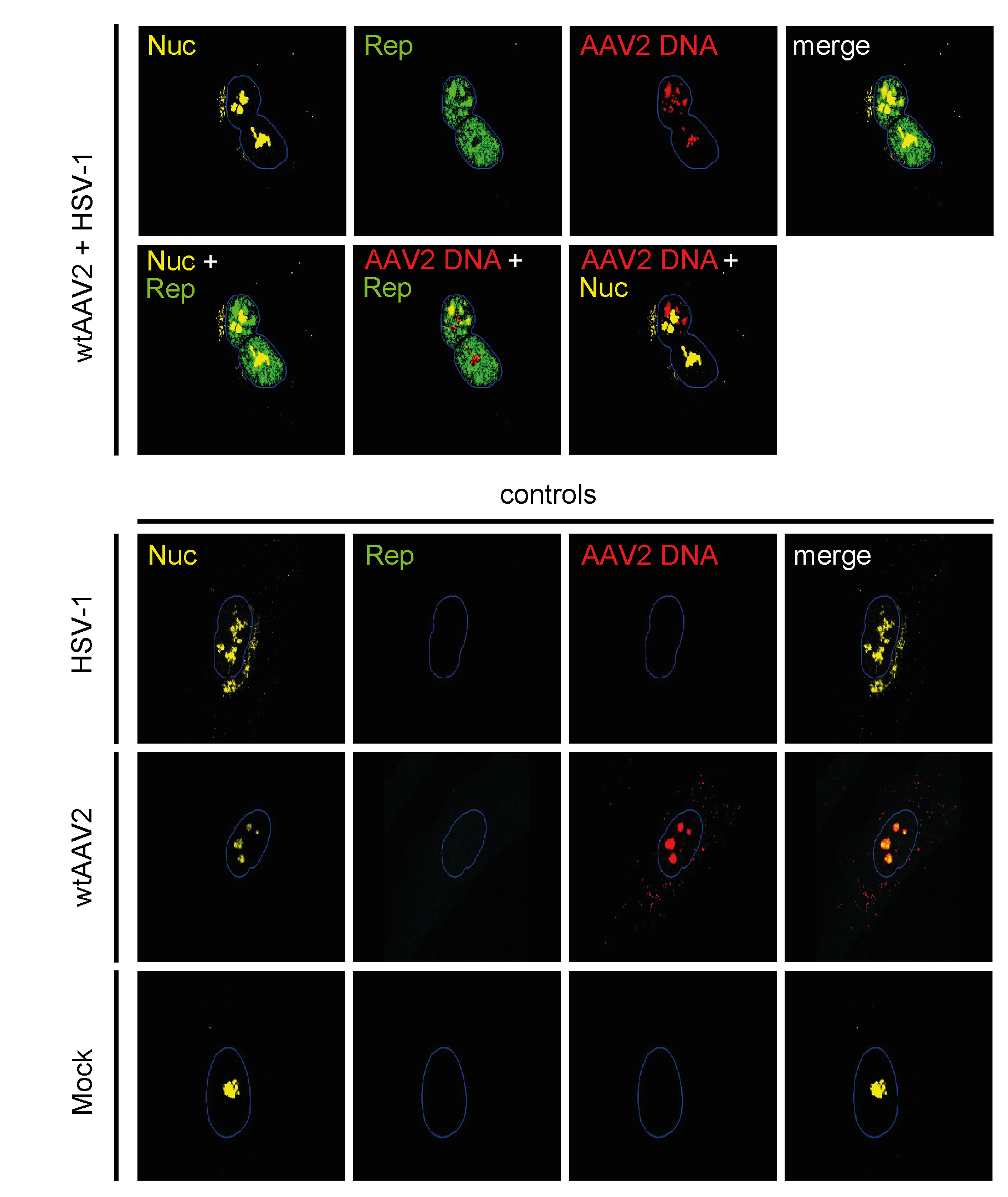

Supplement: S14 Fig — NHF cells were mock-infected or infected with wtAAV2 (MOI 10`000), HSV-1 (MOI 0.5) or co-infected with wtAAV2 (MOI 5`000) and HSV-1 (MOI 0.5). At 12 hpi, the cells were fixed and processed for multicolor IF analysis combined with FISH and CLSM. Nucleoli (Nuc) were visualized using an antibody against fibrillarin (yellow). wtAAV2 replication compartments were stained using a primary antibody specific for the AAV2 Rep proteins (green). AAV2 DNA (red) was detected with an Alexa Fluor (AF) 647 labeled, amine-modified DNA probe that binds to the AAV2 genome. Nuclei were counterstained with DAPI and illustrated as blue lines. (TIF) [file ppat.1010187.s014.tif]
