# Supplementary material for: Adeno-associated virus type 2 (AAV2) uncoating is a stepwise process and is linked to structural reorganization of the nucleolus
Source: PLoS Pathog. 2022 Jul 11;18(7):e1010187. doi: 10.1371/journal.ppat.1010187 (PMC9302821; doi:10.1371/journal.ppat.1010187)
Supplement: Attachment — Submitted filename: Rebuttal letter_final.docx [file ppat.1010187.s016.docx]

Dear Dr. Weitzman,

Dear Dr. Münger,

Dear Dr. Haldar,

Dear Dr. Malim,

We would like to thank you and the reviewers for the critical assessment of our manuscript and for giving us the opportunity to submit a revised version. Based on the reviewers’ suggestions, we have performed several additional experiments and included new data. Importantly, experiments to assess the functionality of the AAV2 DNA released into the nucleoli.

Please find our responses to the specific issues below.

**Part I – Summary**

Reviewer #1: Since the first observations of the presence of AAV capsids within the nucleolus, several studies have shown this nuclear body interacts with AAV at both early and late steps of the viral life cycle, a notion reinforced by finding that AAV capsids interact with nucleolin and nucleophosmin (B23). However, while the nucleolus is believed to favor capsid assembly during the late stage of infection, its function during the early infectious steps is still unclear. In particular, previous studies conducted with recombinant AAV vectors have suggested that the nucleolus may sequester AAV capsids upon nuclear entry and that capsid disassembly and genome uncoating requires their translocation in the nucleoplasm.

The aim of this study was to revisit this notion using immuno-FISH analyses to detect the release of the wt AAV2 genome together with the viral capsids and/or VP proteins upon infection of human primary fibroblasts. The results presented by Sutter and colleagues show that wt AAV2 single-stranded DNA can be detected in the nucleolus of the infected cells either associated to assembled AAV2 capsids or AAV2 VP proteins. Further, the authors show data that remodeling of the nucleolar structure, a phenomenon known to occur upon cell division, favors capsid disruption and release of AAV2 DNA. They conclude from these data that uncoating of AAV2 occurs in the nucleolus upon its structural reorganization during cell cycle.

While the analyses performed by Immuno-FISH are well-conducted and informative, the whole study suffers from the lack of functional analyses to support the conclusion. In particular, the absence of information about the functionality of the viral DNA released into the nucleolus, in terms of capacity to establish as a stable episome and to initiate viral replication makes any conclusion about this process premature.

We would like to thank Reviewer #1 for the thorough assessment of our work and the helpful suggestions to improve it. We have now addressed the question concerning the functionality of the nucleolar AAV2 DNA in two additional experiments. The results can be summarized as follows: (I) AAV2 DNA released into the nucleoli (bead-purified nucleolar DNA) is transcriptionally active upon transfection (S6 Fig; lines 258-261); (II) we identified AAV2 replication form monomers (rfm), the product of second-strand synthesis, in nucleoli (Fig. 5; lines 261-268, 549-553). The last finding is based on Southern analysis of DNA isolated from nuclear, nucleoplasmic, and nucleolar fractions. While we did not observe circular episomes (not shown) up to 48 h of AAV2 infection, we did observe single-stranded (ss) AAV2 DNA rfm DNA with the following kinetics and distribution: ssDNA was clearly visible in the nucleolar fraction by 24 h of infection. In the nucleoplasmic fraction the ssDNA becomes clearly visible not until 48 h of infection. Most interestingly, rfm DNA was observed in the nucleolar fraction both at 24 h and 48 h after infection. The rfm DNA was not observed at either time point in the nucleoplasmic fraction, although at 48 h after infection, the band representing the ss DNA had a comparable intensity in the nucleoplasmic and nucleolar fractions (Fig 5 and lines 261-268, 549-553). Cell fractionation was controlled by Western blot and immunofluorescence/FISH analyses (S5 Fig, lines 256-258).

Reviewer #2: Sutter et al., have provided a body of data supporting a stepwise manner of AAV uncoating and linked to structural reorganization of the nucleolus. Utilizing immunofluorescence analysis and in situ hybridization the authors attempt to tract and segregate the AAV capsid vs AAV DNA genome after infection in vitro. It is very well written, and reads like an unraveling detective story, where the authors are following the clues (data that emerged from one experiment) to inform additional experiments to refine and expand their hypothesis. The strength and the novelty of the studies reside in the author’s ability to follow the process of AAV2 infection and uncoating at a single cell resolution, therefore providing a unique glimpse into AAV2 biology. A potential weakness is the number of particles utilized in order to obtain a convincing signal for imaging. It would be prudent if the authors could compare the doses utilized to capture the data in their experiments with published data studying the infectious MOI required to obtain transduction. The potential risk of saturating pathways with virus particles in order to observe an image should be discussed. Regardless, the authors performed robust experiments, and provide conservative interpretation.

One major missing piece is the lack of discussion on how those observations affect the utility of AAV vectors for gene therapy medicines. AAV vectors are used widely to develop gene therapy vectors for where drug potency is dependent on the ability of the vectors to transduce non-dividing cells in a host. One suggestion is to include a discussion on how these data may be relevant for our understanding of how gene therapy works/may be improved. This work was done using wild type AAV2. Are those observations specific only to wild type AAV2? The authors should comment on this point. Do the authors expect that recombinant AAV vectors may behave differently? Please address in discussion. Do the authors expect that different AAV serotypes will be similar to the wild type AAV2? If such discussion is included, this position the manuscript to be of broader interest.

Minor concern

Reference 25 is repeated as ref 28. It appears the authors have inadvertently used the same ref with two different numbers, either remove and change the numbering or replace with correct reference.

We would like to thank Reviewer #2 for the positive feedback and important suggestions to make our work of broader interest.

The relatively high MOI of 20’000 gcp/cell was chosen to facilitate easy detection of AAV2 capsids and DNA on the single cell level. The chosen MOI is in line with previous studies from other groups facing the same challenge of sensitivity (e.g., Junod et al. 2021, DOI: 10.3390/v13020167: 10^4^ per cell; Nonnenmacher and Weber 2014, DOI: 10.1016/j.chom.2011.10.014: 10^5^ per cell). However, such high MOIs may indeed affect virus trafficking. We have now performed an experiment with a 10-fold lower MOI (2000 gcp/cell) and obtained comparable results concerning uncoating in the nucleoli. The data is shown in S1 Fig and described in lanes 141-145 of the revised manuscript.

In the revised manuscript we also discuss the potential impact of our findings for AAV vector-mediated gene therapy (lines 594-602). In addition, we performed additional IF-FISH experiments and show nucleolar accumulation and uncoating also in cells infected with a recombinant AAV vector (rAAVGFP; S2 Fig and lines 141-145).

We focused on AAV serotype 2 (AAV2) as the so far best characterized serotype. Serotypes differ in epitopes recognized by the host’s immune system and in receptors used for cell entry (Wang et al., 2019, DOI:10.1038/s41573-019-0012-9; Macdonald et al., 2021, DOI:10.1089/hum.2021.169). In addition, variations in capsid stability and in the efficiency of uncoating and episome formation have been described (Penzes et al., 2021, DOI:10.1128/JVI.00843-21; Nam et al., 2011, DOI:10.1128/JVI.05305-11; Wang et al., 2007, DOI:10.1073/pnas.0702778104). These analyses are based on AAV serotype vectors not viruses. Despite these differences and despite the existence of AAV serotypes that transduce cells independent of AAVR and/or GPR108 (Dudek et al., 2020, DOI:10.1016/j.ymthe.2019.11.005), all serotypes follow the same general steps for cell transduction differing mainly in the efficacy by which the different steps are mastered. We therefore expect that the uncoating process is similar for all serotypes. This hypothesis needs to be confirmed experimentally. This, however, exceeds the topic and focus of our current study.

The issue with references 25 and 28 has been resolved.

Reviewer #3: The study by Sutter et al is focused on dissecting capsid uncoating steps in the infectious pathway of adeno-associated virus serotype 2 in mammalian cells. This step is critical for subsequent events in the AAV life cycle such as second strand synthesis and transcription. The study is technically sound and the authors skillfully utilize fluorescence imaging techniques and quantitation to dissect subtle shifts in AAV biology under different pharmacologically manipulated host cell conditions. Review of the existing literature and data interpretation are woven together eloquently and the overall manuscript is well written. Major strengths lie in the technical execution and quality of the study. Significant weaknesses are identified with regard to study parameters and overall descriptive nature of the findings. No significant new mechanistic insight is gleaned from the data. Major concerns include:

We would like to thank Reviewer #3 for the critical assessment of our work and for the important suggestions. Uncoating is a major step in the life cycle of a virus and difficult to assess. In addition, understanding the mechanism of uncoating also impacts on AAV vector-mediated gene therapy. Our study, for the first time, investigated AAV2 uncoating on the single cell level. We provide strong evidence that uncoating takes place in a stepwise process that is completed in the nucleolus. While much of the data is descriptive, our work also reveals that uncoating is enhanced by changes in the nucleolar structure during the cell cycle. While the underlying molecular mechanism remains to be elucidated, the finding that the cell cycle is involved provides important hints on the mechanism.

1. The use of fibroblasts as a benchmark for studying AAV biology is a concern. In the gene therapy context, it is well understood that most AAV serotypes suffer significant bottlenecks in the infectious pathway(s) pertinent to fibroblasts (e.g. inefficient endosomal trafficking pathways and proteasomal degradation). It is unlikely that the findings are pertinent to AAV capsid biology in general or in the context of pertinent cells such as neurons, cardiomyocytes or hepatocytes as examples.

We are grateful for the review for bringing up this concern. In response, we have performed IF-FISH analysis with wtAAV2 on a set of different cell types. Specifically, wtAAV2 infected lung and kidney epithelial cells (A549 and Vero, respectively), and in rAAVGFP infected hepatocytes (Hep G2), normal human fibroblasts (NHF), and lung epithelial cells (A549). The nucleolar accumulation of AAV2 capsids and uncoating of AAV2 DNA was comparable in all cell lines and with both wild-type and recombinant AAV2 (S2 Fig and lines 141-145).

2. The immunofluorescence based image analysis raises several significant concerns. First, the A20 antibody against the intact AAV2 capsid does not appear to recognize the capsid following conformational changes upon nuclear entry (e.g., multiple J Virol studies from Kleinschmidt, Asokan, Muzyczka and other groups). Thus the lack of A20 signal is not indicative of fully uncoated capsids. In addition, the A1/A69 antibodies against the VP1/2 domains exhibit high levels of background/cross-reactive staining in IF studies and are more reliable for western blot analysis. Thus, the use of fluorescently labeled AAV capsids is generally more reliable (e.g., Xiao and Samulski, Johnson and Samulski). In particular, the ability to recover fully infectious AAV particles purified from nucleoli post-infection has been reported (Johnson and Samulski, 2009).

Again, we are grateful for the reviewer to address these concerns. In our study, the A20 antibody clearly recognizes AAV2 capsids in the nucleus and nucleolus (see e.g., Fig 1). Nuclear and nucleolar capsid signals were detected also in several other studies including Wistuba et al., 1997, DOI: [10.1128/JVI.71.2.1341-1352.1997](https://doi.org/10.1128/jvi.71.2.1341-1352.1997) ; Sonntag et al., 2010, DOI: [10.1073/pnas.1001673107.](https://doi.org/10.1073/pnas.1001673107)  Also, in the study by Johnson and Samulski 2009, DOI: [10.1128/JVI.02309-08](https://doi.org/10.1128/jvi.02309-08) , mentioned by the reviewer, A20 was used to detect intact capsids.

Furthermore, while we used A69 (not A1) for a single experiment, we have used VP51 (B1) antibody for most experiments. In none of our experiments, we have observed a problem with high levels of background/crossreactive staining with any of the antibodies.

We also would like to point out that the finding of infectious AAV particles from nucleoli – described in the Johnson and Samulski study - is not contradicting our findings in any way.

3. Several key steps identified such as the need for endosomal escape, effects of BafA1 on AAV2 infection have been extensively mapped by other groups, these findings do not provide any new/additional insight. Further, the impact of proteasomal inhibitors, hydroxyurea etc on the fate of AAV in the nucleus have been reported in significant detail (e.g., Johnson and Samulski). This is especially critical for the authors to consider since drugs such as Actinomycin D disrupt nucleolar organization, while proteasome inhibitors leave the nucleolus intact (both can be utilized to block transcription). Yet, both appear to result in increased infectivity/transduction. This dichotomy needs to be resolved.

We have performed these experiments to show that step 1 of the uncoating process (genome accessibility for the FISH probe in A20 positive capsids) is supported by acidification and not to show the need for endosomal escape. We used Actinomycin D exactly because we wanted to perturb the nucleolar structure and to assess its role in AAV2 DNA release and disappearance of intact capsid signal.

4. Lastly, while the findings related to cell cycle impact on AAV-host nucleus interactions are interesting, little mechanistic insight can be gleaned from these findings. Are there nucleolar factors that mediate AAV capsid uncoating? For instance, Nicolson and Samulski show the need for nuclear import factors, while Johnson and Samulski show that similar to effects observed with proteasome inhibition, siRNA-mediated knockdown of nucleophosmin potentiated nucleolar accumulation and increased transduction 5- to 15-fold. Parallel to effects from hydroxyurea, knockdown of nucleolin mobilized capsids to the nucleoplasm and increased transduction 10- to 30-fold. A somewhat related concern that follows is that since wtAAV is being utilized, the authors do not provide transduction data. This is a critical missing piece of mechanistic data, since it is unclear whether disrupting the nucleolus/cell cycle influences uncoating and consequently, infectivity/transduction. For instance, how would the authors deconvolute the interplay between Thymidine and ActD treatments influence infectivity/transduction given ActD mediates both nucleolar reorganization and blocks transcription?

To address the question how proteasome inhibitors affect the AAV2 capsid-positive, DNA-positive (AAV2 capsid+DNA+) signal, NHF cells were either mock-infected or infected with wtAAV2 (MOI 20`000) in presence or absence of the proteasome inhibitor (MG132). The drug treatment was performed as described by Johnson and Samulski, 2009 (DOI: 10.1128/JVI.02309-08). The samples were fixed at different time points post infection and processed for combined IF-FISH.

At 16 hours post infection hpi, we observed a peak in the accumulation of AAV2 capsid+DNA+ signals in the nucleoli of AAV2 infected cells. The MG132 treatment, however, started to affect the nucleolar structure (forming nucleolar caps) after 16 h (see **Figure MG132 treatment** below).

**Figure MG132 treatment.** NHF cells were treated with MG132 (2 µM) during the time of infection with wtAAV2 (MOI 20`000). Different time points post infection, the cells were fixed and processed for

combined IF-FISH and CLSM. Nucleoli (Nuc) were visualized using an antibody against fibrillarin (yellow). Intact capsids were stained using an antibody (A20) that detects a conformational capsid epitope (green). AAV2 DNA (red) was detected with an Alexa Fluor (AF) 647 labeled, amine-modified DNA probe that binds to the AAV2 genome. Nuclei were counterstained with DAPI and illustrated as white lines.

We also assessed the effect of the post-transcriptional silencing of NCL and NPM1 (B23) genes on the AAV2 capsid+DNA+ vs. AAV2 capsid-DNA+ signal. Specifically, NHF cells were transfected with no siRNA, scrambled (SCR) control siRNA, or different siRNAs targeting NCL or NPM1 (target sequences were adapted from Johnson and Samulski, 2009). 40 hours post transfection (hpt), cells were either mock-infected or infected with wtAAV2 (MOI 20`000) and 24 h later processed for combined IF-FISH and CLSM (see **Figure Post-transcriptional silencing of NCL and NPM1** below). The IF-FISH was performed as described in the materials and methods section of the revised manuscript. Total cell lysates prepared at 40 hpt were subjected to Western analysis to confirm the knock-down of NCL or NPM1. Vimentin was used as loading control.

Overall, the IF-FISH experiments showed that nucleoli disintegrated and the AAV2 DNA signal consequently localized to the nucleoplasm upon post-transcriptional silencing of NCL. While this finding is in accordance with the study of Johnson and Samulski (2009), we did not observe the nucleolar accumulation of AAV2 capsids upon knock-down of NPM1 because in our experiment, the post-transcriptional silencing of NPM1 also resulted in the disintegration of the nucleoli and the localization of the AAV2 DNA signal in the nucleoplasm. This may be a cell type specific issue.

**Figure Post-transcriptional silencing of NCL and NPM1.** NHF cells were transfected with no siRNA, scrambled (SCR) control siRNA, or different siRNAs targeting NCL or NPM1 (target sequences were adapted from Johnson and Samulski, 2009). 40 hours post transfection (hpt), cells were either mock-infected or infected with wtAAV2 (MOI 20`000) and 24 h later processed for combined IF-FISH and CLSM. (A) Intact capsids were stained using an antibody (A20) that detects a conformational capsid epitope (green). Nucleoli (Nuc) were visualized using an antibody against fibrillarin (yellow). AAV2 DNA (red) was detected with an Alexa Fluor (AF) 647 labeled, amine-modified DNA probe that binds to the AAV2 genome. Nuclei were counterstained with DAPI. (B) Knock-down of NCL and NPM1 was confirmed on protein level.

Concerning transduction, we have now performed also uncoating/transduction experiments using a recombinant AAV vector. These experiments reveal the nucleolar accumulation and uncoating also in cells infected with a recombinant AAV (rAAVGFP) and show that transduction correlates with complete uncoating in the nucleoli (S2 Fig and lines 141-145). See also response to Reviewer #2 above.

Reviewer #4: The manuscript by Sutter et al. addresses the question how Adeno-associated virus type 2 (AAV2) fully uncoat and liberate their genome in the nucleus of infected cells. This is an important and timely topic because AAVs are increasingly and successfully used as gene therapy vectors. Knowledge that improves our understanding of AAV transduction thus may influence vector development or application. In addition, how virus uncoating is achieved upon cell entry is still not very well known.

The presented work uses quantitative single cell imaging analysis to investigate the subcellular distribution of intact AVV2 capsids, capsid proteins (for disassembled virions) and AVV2 genomes (using FISH analysis for free or partially exposed genomes) over time and under different physiological conditions. They find that AAV2 virions partially exposing the viral genome can be found in the cytosol and accumulate over time in the nucleolus. They further show that the nucleolus is also the place where AAV2 virions fully uncoat, probably in a manner that involves structural and/or functional reorganization of the nucleolus.

This work is technically well executed and the data presentation is mostly clear. However, the conclusions that can be drawn are limited by the descriptive nature of the data and I struggle at times to follow the authors line of argument. The uncoating mechanism in the nucleolus is not addressed.

We would like to thank Reviewer #4 for the positive feedback and the suggestions to improve the work. We agree that much of the data is descriptive. However, uncoating is a key event in the virus life cycle that is difficult to investigate. We believe that our work makes a significant contribution to understanding this process. While the underlying molecular mechanism remains to be elucidated, the finding that the cell cycle is involved provides at least some mechanistic insight.

I have the following comments;

1- The quantification in Fig. 1B/C indicates that the ratio of partially uncoated particles (capsid+DNA+) increases in the cytosol as well as in the nucleus over time. How was this ratio determined as I find it hard to identify individual capsids/DNA signals especially in the nucleolus at later time points?

We have used CellProfiler which assesses primary objects based on diameter in pixel units. The overlaps of two primary objects (capsid and DNA) results in capsid+/DNA+ counts.

2- The authors argue that their data show a partial disassembly step taking place in the cytosol (line 473-476), which requires acidification (Fig3). Is the bafilomycin treatment not rather affecting AAV2 uptake/endosome penetration/escape (bafilomycin treated cells seem to have less overall cell associated virions in the examples shown)? I am also surprised to see that the endosome specific 76HD/AN mutant shows large scale partial disassembly (Fig. S1), which in my opinion argues more for an intra endosomal/endsome penetration associated partial disassembly. Could you please comment why you think this is a cytosolic step? Please provide experimental evidence for a cytosolic localization of the partial disassembled virions.

We entirely agree. Our data support the conclusion that the partial disassembly step takes place in the cytoplasm, likely within transport vesicles. We do not claim partial uncoating in the cytosol. We clarified this issue in lines 525, 200-2001.

3- How are the two observed virion pools (capsid+DNA+ vs. capsid+DNA-) in the two compartments (nucleus and cytosol) functionally connected? I wonder if the partially disassembled pool in the cytosol contributes to the nucleolar pool. If this is the case are the authors suggesting that both, partially disassembled virions (capsid+DNA+) and intact virions (capsid+DNA-) can be nuclear import substrates as both forms are present in the cytosol as well as in the nucleolus? Would this not imply that partial disassembly can also take place in the nucleolus, especially in light of their findings that all virions fully uncoat in the nucleolus?

We agree that at this resolution we cannot completely exclude the possibility that entirely intact capsids get access to the nucleolus. However, the exposure to acidification in endocytic vesicles, resulting in capsid+DNA+ entities according to our data, has been considered to be essential for nuclear entry (e.g., Sonntag et al., 2006, DOI: 10.1128/JVI.01056-06; Johnson et al., 2010, DOI: 10.1128/JVI.00687-10).

4- The observation of some cells displaying nucleoli full of genomes but devoid of virions (Fig.4) and of cells with VP1/2/3 specific signals but absence of virion signals (Fig.5) is intriguing. Fig4 and 5 should be redone as time point experiment in cycling cells and the phenotypes should be quantified. One would expect that if the phenotypes I-III are consecutive steps that this is reflected over time and the number of capsids should diminish and the VP1/2/3 signal should increase. A similar kinetic in resting cells or contact inhibited cells should result in an uncoating defect.

We have performed time point experiments comparing AAV2 uncoating in cycling cells (40% confluency) and contact inhibited cells (100% confluency). In the cycling cells, complete uncoating increased over time, while in growth arrested (contact inhibited) cells, a complete uncoating defect was observed. See S12 Fig and lines 403-411.

5- Fig. 8 Why is there so much more DNA in the cytosol when using ActD (Fig.8A)? Would that also be the case in absence of the Thymidine block? How was the nucleolar area determined if ActD treatment redistributes the marker?

The reason for the increased DNA signal within the cytoplasm might be due to the ActD-mediated induction of the autophagic flux (Cortes et al., 2016, DOI: [10.1186/s12943-015-0489-8](https://dx.doi.org/10.1186%2Fs12943-015-0489-8)), thereby accelerating the formation of acidic vesicles and resulting in enhanced accessibility of the AAV2 genome for the AAV2 DNA specific FISH probe. As ActD treatment results in nucleolar segregation and nucleolar cap formation we used the DAPI staining which indirectly reveals the position of the nucleoli. More specifically, we segmented the putative nucleolar area based on the missing pixel units of DAPI in CellProfiler.

**Part II – Major Issues: Key Experiments Required for Acceptance**

Reviewer #1:

-Fig.1: the observations by immuno-FISH should be correlated with a kinetic analysis of the accumulation of viral DNA (total and packaged) as well as the capsids in the cytoplasm/nucleoplasm and nucleolus after cell fractionation . It would be also important to know if any viral RNA can be detected. Indeed, even if wt AAV2 cannot replicate in the absence of helper virus, a low level of Rep RNA may be produced, at least at the onset of infection.

We have performed a kinetic analysis of AAV2 DNA accumulation in nuclear, nucleolar, and nucleoplasmic fractions at 24 h and 48 h of infection by Southern blot. Single-stranded AAV2 DNA was clearly visible in the nucleolar fraction by 24 h of infection. In the nucleoplasmic fraction the ssDNA becomes clearly visible not until 48 h of infection. Most interestingly, rfm DNA, the product of second-strand synthesis, was observed in the nucleolar fraction both at 24 h and 48 h after infection. The rfm DNA was not observed at either time point in the nucleoplasmic fraction, although at 48 h after infection, the band representing the ssDNA had a comparable intensity in the nucleoplasmic and nucleolar fractions (Fig 5 and lines 261-268). Cell fractionation was controlled by Western blot and immunofluorescence/FISH analyses (S5 Fig, lines 256-258).

An obvious next question is whether AAV2 DNA is transcribed in the nucleoli. However, we prefer to investigate this possibility in a follow-up study and to keep the focus on uncoating.

- Fig.1: several AAV DNA-positive/capsid-negative spots are visible in the cytoplasm of the cells. Are these signals still visible after treatment of cells with nucleases (DNase or RNAse)? And how does this treatment affect the capsid-positive/DNA-positive signals?

To address the question how nuclease treatments affect the AAV2 capsid-positive, genome-positive (AAV2 capsid+DNA+) signal, NHF and A549 cells were either mock-infected or infected with wtAAV2 (MOI 20`000) and 24 h later processed for combined IF-FISH and CLSM (S4 Fig and line 155). The IF-FISH was performed as described in the materials and methods section of the revised manuscript, except that wtAAV2 infected samples were either treated with DNase I (1 U/µl) for 1 h at 37°C, RNase A (0.5 mg/ml) for 1 h at 37°C, or a combination of both. Overall, the experiments showed that the complete AAV2 genome signal disappeared in both cell lines after DNase I -, or DNase I plus RNase A treatment but not upon RNase A treatment alone. It should be noted that treating NHF cells with DNase I led to reduced DAPI signal and a slightly stronger background level probably due to cellular autofluorescence. Both cell lines were recorded with identical settings to allow the comparison between the two different cell lines.

- Fig.4: does the capsid-negative/AAV DNA-positive signal increase over time?

We have now performed time point experiments and show that complete AAV2 uncoating increased over time. See also response to issue 4 of Reviewer #4 and S12 Fig, lines 403-411.

- Release of viral DNA could occur by a physiological uncoating process or following disruption of the capsids, both mechanisms not necessarily resulting in the same outcome in terms of viral establishment. Does the capsid-negative nucleolar AAV2 DNA signal correlate with the formation of double-stranded circular episomes?

We have addressed this issue using cells infected with a recombinant AAV2 vector (rAAVGFP). The results are shown in S2 Fig and show that the transduction efficiency (% GFP positive cells) correlates with the uncoating efficiency (% rAAV DNA-positive and AAV capsid-negative cells; (S2 Fig; lines 141-145). Moreover, we have investigated the presence of AAV2 replication intermediates in nuclear, nucleolar, and nucleoplasmic fractions. Specifically, we have analyzed DNA isolated from the different cellular fractions in presence or absence of T5 nuclease, which degrades nucleic acid species that are not double-stranded and circular, by Southern blot using a AAV2 rep-specific probe. No bands were detected in the T5 traeted samples, indicating that no circular episomes were present in any of the fractions at least until 48 h of infection. However, the non T5 nuclease treated samples revealed the following kinetics and distribution of AAV2 DNA: ssDNA was clearly visible in the nucleolar fraction by 24 h of infection. In the nucleoplasmic fraction the ssDNA becomes clearly visible not until 48 h of infection. Most interestingly, rfm DNA, the product of second-strand synthesis, was observed in the nucleolar fraction both at 24 h and 48 h after infection. The rfm DNA was not observed at either time point in the nucleoplasmic fraction, although at 48 h after infection, the band representing the ssDNA had a comparable intensity in the nucleoplasmic and nucleolar fractions (Fig 5 and lines 261-268 and 249-253). Cell fractionation was controlled by Western blot and immunofluorescence/FISH analyses (S5 Fig, lines 256-258).

- Proteasome inhibitors have been shown to increase the accumulation of capsids in the nucleolus as well as transduction with rAAV vectors. What is the effect of this treatment on the FISH signals detected in the nucleolus?

To address the question how proteasome inhibitors affect the AAV2 capsid-positive, genome-positive (AAV2 capsid+DNA+) signal, NHF cells were either mock-infected or infected with wtAAV2 (MOI 20`000) in presence or absence of the proteasome inhibitor (MG132). The drug treatment was performed as described by Johnson and Samulski, 2009 (DOI: [10.1128/JVI.02309-08).](https://doi.org/10.1128/jvi.02309-08) The samples were fixed at different time points post infection and processed for combined IF-FISH.

At 16 hpi, we observed a peak in the accumulation of AAV2 capsid+DNA+ signals in the nucleoli of AAV2 infected cells. The MG132 treatment, however, started to affect the nucleolar structure (forming nucleolar caps) after 16 h. See also response to Reviewer #3 above and Figure **MG132 treatment** included therein).

- Similarly, previous studies have shown that siRNA against nucleolin and B23 alter the translocation of the capsids from the nucleolus to nucleoplasm and transduction with AAV vectors. How do these treatments affect the presence of capsid-negative AAV2 DNA in the nucleolus and the formation of double-stranded circular episomes?

We assessed the effect of the post-transcriptional silencing of NCL and NPM1 (B23) genes on the AAV2 capsid+DNA+ vs. AAV2 capsid-DNA+ signal. Specifically, NHF cells were transfected with no siRNA, scrambled (SCR) control siRNA, or different siRNAs targeting NCL or NPM1 (target sequences were adapted from Johnson and Samulski, 2009). 40 hours post transfection (hpt), cells were either mock-infected or infected with wtAAV2 (MOI 20`000) and 24 h later processed for combined IF-FISH and CLSM. The IF-FISH was performed as described in the materials and methods section of the revised manuscript. Total cell lysates prepared at 40 hpt were subjected to Western analysis to confirm the knock-down of NCL or NPM1. Vimentin was used as loading control.

Overall, the IF-FISH experiments showed that nucleoli disintegrated and the AAV2 DNA signal consequently localized to the nucleoplasm upon post-transcriptional silencing of NCL. While this finding is in accordance with the study of Johnson and Samulski (2009), we did not observe the nucleolar accumulation of AAV2 capsids upon knock-down of NPM1 because in our experiment, the post-transcriptional silencing of NPM1 also resulted in the disintegration of the nucleoli and the localization of the AAV2 DNA signal in the nucleoplasm. This may be a cell type specific issue. See also response to Reviewer #3 above and Figure **Post-transcriptional silencing of NCL and NPM1** included therein.

- Since the authors are using primary fibroblast, growth arrested cells should be used instead of artificially-arrested cells to examine the fate of the capsids/VP proteins and viral DNA in the nucleolus of non-dividing cells. This would also allow to keep the cells in culture for longer periods to perform kinetic analyses.

We have performed time point experiments comparing AAV2 uncoating in cycling cells (40% confluency) and contact inhibited cells (100% confluency). In the cycling cells, complete uncoating increased over time, while in growth arrested (contact inhibited) cells, a complete uncoating defect was observed. See S12 Fig and lines 403-411; see also Reviewer #4.

Reviewer #2: No major issues

Reviewer #3: N/A

Reviewer #4: - Please provide experimental evidence that partial disassembly of virions (capsid+DNA+) occurs in the cytosol or change the statement

We clarified that partial uncoating takes place in the cytoplasm, likely in endocytic vesicles (lines 525, 200-2001).

- Experiments in Fig4 and 5 should be redone as time point experiment in cycling cells and the phenotypes should be quantified.

We have performed time point experiments comparing AAV2 uncoating in cycling cells (40% confluency) and contact inhibited cells (100% confluency). In the cycling cells, complete uncoating increased over time, while in growth arrested (contact inhibited) cells, a complete uncoating defect was observed (S12 Fig and lines 403-411).

**Part III – Minor Issues: Editorial and Data Presentation Modifications**

Reviewer #1: - Lines 82/94/455 References to previous studies showing accumulation of AAV capsid in the nucleolus are not appropriate. The references cited by the authors (Ref.14 and 15) refer to studies showing the interaction of the capsid with nucleolin and B23. But several previous studies had

documented the presence of capsid in the nucleolus.

We would like to apologize that our phrasing was not appropriate. We corrected this issue in lines 83 and 95.

- Fig.3: why inhibition with Bafilomycin is not complete?

We adapted the maximum used concentration of bafilomycin A1 (200 nM in HeLa cells) from Bartlett et al., 2000 (DOI: [10.1128/jvi.74.6.2777-2785.2000](https://doi.org/10.1128/jvi.74.6.2777-2785.2000)). In our study, however, we treated the cells (NHF cells) only for 1 h prior to infection and not during the entire infection, which may have resulted in an incomplete inhibition.

- Fig.3C and line 199: nuclear or nucleolar AAV2 import or counts? Why counting only capsid-positive signals and not, as before capsid-positive/DNA-positive signals?

We performed this experiment to validate the IF-FISH assay, as it is known from the literature that bafilomycin A1 treatment prevents acidification, endosomal escape, and nuclear entry (e.g., Sonntag et al., 2006, DOI: 10.1128/JVI.01056-06; Johnson et al., 2010, DOI: 10.1128/JVI.00687-10). Indeed, bafilomycin A1 strongly reduced the nuclear AAV2 capsid- and DNA signals (Fig 3A), thereby confirming the specificity of the stainings. For the quantification of the nuclear entry of AAV2 capsids as shown in Fig 3C, the AAV2 DNA signal was not relevant.

- Fig8A: actinomycin D treatment seems to equally increase the AAV2 DNA signal in cytoplasm. How do the authors explain this? Actinomycin D can inhibit the intracellular transport of viral capsids as shown for Influenza virus. Could this explain the lower AAV2 capsid signal observed in the nucleolus?

The increased DNA signal within the cytoplasm might be due to the Actinomycin D-mediated induction of the autophagic flux (Cortes et al., 2016, DOI: [10.1186/s12943-015-0489-8](https://dx.doi.org/10.1186%2Fs12943-015-0489-8)), thereby accelerating the formation of acidic vesicles and resulting in enhanced accessibility of the AAV2 genome for the AAV2 DNA specific FISH probe. See also response to Reviewer #4 above.

The lower AAV2 capsid signal in the nucleolus cannot be explained by a potential inhibition of intracellular transport because Actinomycin D treatment was performed for 1 h at 24 h after infection, a time point where nucleolar accumulation has already taken place (see Fig 1).

-Fig.8A: despite the low level of fibrillarin staining, nucleoli are still visible by DAPI staining. Please explain.

Fibrillarin is part of the dense fibrillary compartment and the fibrillary center. Actinomycin D induces the formation of nucleolar caps (L. Latonen, 2019, DOI: 10.3389/fncel.2019.00151) resulting in the vanishing of fibrillarin. However, actinomycin D does not induce complete nucleolar necklace and the nucleolar remnants remain visible in the DAPI staining (see Figure 2 of r L. Latonen, 2019, DOI: 10.3389/fncel.2019.00151).

- Line 473: uncoating or just remodeling? Extrusion of the VP1/2 N-ter may provide access to small DNA probes or simply labeled nucleotides.

We agree that we cannot distinguish between remodeling or protrusion of the AAV2 DNA within the cytoplasm. Therefore, we defined the cytoplasmic observation of AAV2 capsid+DNA+ signals as step 1 of the uncoating process and designated it as genome accessibility (e.g. line 158).

- FigS2 and line 250: a negative correlation between capsid (A20) signal and individual VP staining is not evident in this image. Please explain.

Our phrasing was not appropriate. We corrected this issue by changing the title of that section lines 292-293 and 300 of the revised manuscript.

- Fig.S5: decrease of cells in G1 and/or increase in G2 upon nocodazole treatment is much less evident by flow cytometry than CLSM. Please explain.

The samples were processed slightly different; for flow cytometry the cells were harvested, fixed, and directly stained with DAPI. For CLSM the cells were embedded in ProLong Anti-Fade mountant containing DAPI. Also, the gating approach is different between flow cytometry and CLSM.

- line 529: The statement that AAV vectors do not efficiently transduce non-dividing cells is not correct. Transduction of non-dividing by AAV vectors can efficiently occurs even in vitro (provided that the receptor is present) but with a different kinetics than that observed on dividing cells.

We thank Reviewer #1 for this input and clarified this issue in lines 598-600.

Reviewer #2: No minor issues

Reviewer #3: N/A

Reviewer #4: - please note in each legend if the images are confocal midsections or if they are Z-projections. An outline of the nucleus in addition to the nucleolus might help. This is important to appreciate the localization of objects inside/on-top or below of the nucleus.

We thank Reviewer #4 for this input and adjusted each legend in the revised manuscript. Also, we outlined the nucleus as blue or white line in addition to the outline of the nucleolus in Figs 1, 3, and S3.

- Please make sure that Figure numbers are mentioned in the text in the order they appear (e.g. Fig 1C, Fig.4C appear out of order)

We corrected this issue in the revised manuscript.

- Fig9 please label what is shown in top vs bottom line (same for FigS9), graph and graph legend don’t match (light color for VP, no light grey color in graph) (same for FigS9)

Following a similar classification for the capsid staining as applied for Fig 4, we have now assigned the top and bottom panels of Fig 10A (formerly Fig 9A) as pattern I and III. We have applied this classification also in Fig 6 and S7 Fig.

PLOS authors have the option to publish the peer review history of their article ([what does this mean?](https://journals.plos.org/plospathogens/s/editorial-and-peer-review-process#loc-peer-review-history)). If published, this will include your full peer review and any attached files.


If you choose “no”, your identity will remain anonymous but your review may still be made public.


**Do you want your identity to be public for this peer review?** For information about this choice, including consent withdrawal, please see our [Privacy Policy](https://www.plos.org/privacy-policy).

Reviewer #1: No

Reviewer #2: **Yes:** R Jude Samulski PhD

Reviewer #3: No

Reviewer #4: No

Figure Files:

While revising your submission, please upload your figure files to the Preflight Analysis and Conversion Engine (PACE) digital diagnostic tool, https://pacev2.apexcovantage.com. PACE helps ensure that figures meet PLOS requirements. To use PACE, you must first register as a user. Then, login and navigate to the UPLOAD tab, where you will find detailed instructions on how to use the tool. If you encounter any issues or have any questions when using PACE, please email us at figures@plos.org.

Response: All figures were adjusted to the PLOS requirements using PACE.

Data Requirements:

Please note that, as a condition of publication, PLOS' data policy requires that you make available all data used to draw the conclusions outlined in your manuscript. Data must be deposited in an appropriate repository, included within the body of the manuscript, or uploaded as supporting information. This includes all numerical values that were used to generate graphs, histograms etc.. For an example see here:

<http://www.plosbiology.org/article/info%3Adoi%2F10.1371%2Fjournal.pbio.1001908#s5>.

Response: All data is shown in the manuscript, the figures, or the supplementary data.

Reproducibility:

To enhance the reproducibility of your results, we recommend that you deposit your laboratory protocols in protocols.io, where a protocol can be assigned its own identifier (DOI) such that it can be cited independently in the future. Additionally, PLOS ONE offers an option to publish peer-reviewed clinical study protocols. Read more information on sharing protocols at <https://plos.org/protocols?utm_medium=editorial-email&utm_source=authorletters&utm_campaign=protocols>

Response: We use standard protocols, which are widely used and thus we will not deposit our protocols separately.
